# Concurrent Learning of Adjacent and Nonadjacent Dependencies in Visuo-Spatial and Visuo-Verbal Sequences

**DOI:** 10.3389/fpsyg.2019.01107

**Published:** 2019-05-17

**Authors:** Joanne A. Deocampo, Tricia Z. King, Christopher M. Conway

**Affiliations:** ^1^Department of Psychology, Georgia State University, Atlanta, GA, United States; ^2^Neuroscience Institute, Georgia State University, Atlanta, GA, United States

**Keywords:** sequential learning, non-adjacent dependencies, adjacent dependencies, artificial grammar learning, visuo-spatial versus verbal learning

## Abstract

Both adjacent and non-adjacent dependencies (AD and NAD) are present in natural language and other domains, yet the learning of non-adjacent sequential dependencies generally only occurs under favorable circumstances. It is currently unknown to what extent adults can learn AD and NAD, presented concurrently in spatial and verbal sequences during a single session, and whether a second session improves performance. In addition, the relationship between AD and NAD learning and other theoretically related cognitive and language processes has not yet been fully established. In this study, participants reproduced two types of sequences generated from an artificial grammar: visuo-spatial sequences with stimuli presented in four spatial locations, and visuo-verbal sequences with printed syllables. Participants were tested for incidental learning by reproducing novel sequences, half consistent with the grammar and half containing violations of either AD or NAD. The procedure was repeated on a second day. Results showed that both AD and NAD were learned in both visuo-spatial and visuo-verbal tasks, although AD learning was better than NAD and learning of NAD decreased over time. Furthermore, NAD learning for both spatial and verbal tasks was positively correlated with a language measure, whereas AD learning for both spatial and verbal tasks was negatively associated with working memory measures in the opposite domain. These results demonstrate that adults can learn both AD and NAD within a single session, but NAD learning is more easily disrupted than AD and both types of learning are sub-served by partially distinct cognitive processes. These findings increase our understanding of the processes governing the learning of AD and NAD in verbal and spatial domains.

## Introduction

Patterns are a ubiquitous part of life, present all around us in such diverse perception and action domains such as language, music, motor sequencing, visuo-spatial perception, and navigation. A key aspect of human cognition is the ability to learn these patterns that unfold over time across these different domains. This learning ability, referred to as sequential learning or statistical learning (Saffran et al., 1996; Conway and Christiansen, 2001), is essential for mastering skills that require learning, processing, and prediction of proximal or distal occurrences. For example, learning the successive motor patterns of a pitcher may lead a batter to predict the particular trajectory that the ball will take; recognizing the relationship between hearing particular animal calls and the appearance of a predator may allow for avoidance of that predator; and identifying grammatical and semantic dependencies between words and morphemes in a hierarchically arranged sentence may allow the listener to predict subsequent words and keep track of the meaning of the sentence without being distracted by incorrect expectancies. In each of these examples, there may be both adjacent dependencies (AD), that allow for prediction of immediately following items, and non-adjacent dependencies (NAD), that allow for prediction of more distally following items. In many cases, it may be necessary to learn and process both adjacent (proximal) and non-adjacent (distal) contingencies simultaneously and consisting of the same or related stimuli within the same sequence. This is because it may not be possible to separate the two types of dependencies in these real-world situations (e.g., in natural language, any given sentence can contain both AD and NAD simultaneously).

Despite recent interest in understanding NAD learning (e.g., Gómez, 2002; Peña et al., 2002; Creel et al., 2004; Newport and Aslin, 2004; Perruchet et al., 2004; Onnis et al., 2005; Lany et al., 2007; Lany and Gómez, 2008; Pacton and Perruchet, 2008; van den Bos et al., 2012; Romberg and Saffran, 2013; Pacton et al., 2015; Vuong et al., 2016), there is still much we do not know regarding the extent to which individuals can concurrently learn both types of dependencies simultaneously and how learning varies in different domains (e.g., spatial versus verbal), as well as what cognitive processes are recruited for each type of learning. This study attempts to shed light on the nature of AD and NAD learning using visuo-spatial and visuo-verbal sequences and examines the extent to which learning changes over multiple exposure sessions. In addition, this study examines potential associations between each type of dependency learning and various cognitive abilities. Before describing the study in detail, we first review what is currently known about the learning of AD and NAD.

Adjacent dependencies appear to be easily learned from early in infancy (e.g., auditory AD at 8 months: Aslin et al., 1998, and in newborns: Teinonen et al., 2009; visual AD at 2 months: Kirkham et al., 2002, and in newborns: Bulf et al., 2011). Non-adjacent dependencies, however, have been shown to be more difficult to learn, only occurring under special circumstances when the non-adjacent structure is highlighted or when endogenous attention is correctly oriented (de Diego-Balaguer et al., 2016). In one of the first studies intentionally examining NAD learning, Gómez (2002) showed that auditory linguistic AD needs to be made extremely unreliable before infants and adults incidentally noticed the NAD. She did this by making the non-adjacent items in a stream of speech from an artificial language reliably predictive using a simple deterministic rule: A always predicts that B will follow some third intervening item between A and B. On the other hand, the intervening element was highly variable with no predictive value. Infants and adults both learned NAD only when the predictive value of adjacent elements within a sequence was very low. Likewise, in Newport and Aslin (2004), adults only learned contingencies between non-adjacent consonants or vowels when the non-adjacent transitional probabilities were 1.0 but the intervening items were highly variable, making the adjacent relationships non-predictive.

Another way to highlight the existence of NAD is to first train participants on more easily learnable AD and then expose them to sequences of the same predictive pairs but with an intervening item added, transforming the dependencies to non-adjacent ones. Under these extensive training conditions, both infants (Lany and Gómez, 2008) and adults (Lany et al., 2007) were able to learn NAD in auditory artificial languages. Other ways in which auditory NAD has been highlighted involve adding other cues, such as slight pauses between “words” in auditory artificial language word segmentation tasks (e.g., Gómez, 2002; Peña et al., 2002; Perruchet et al., 2004), incorporating additional perceptual cues in other sensory modalities (van den Bos et al., 2012), and making items that are part of a NAD phonologically (Onnis et al., 2005; Frost and Monaghan, 2016) or otherwise perceptually (e.g., Creel et al., 2004) very similar to each other but very different from other items in a sequence. Thus, it appears possible for infants and adults to learn NAD in auditory sequences, but only under these very specialized circumstances.

There has been some evidence of learning of visual or visual-motor NAD in other types of implicit learning tasks as well. For example, Howard and Howard (1997) used a serial reaction time (SRT) task to show that adults responded faster to trials with a repeating non-adjacent pattern, containing a random item (r) interspersed between each item in the pattern (1r2r3r4r1r2r3…), than to trials where all items were random and had no predictive value. Participants implicitly learned this very simple form of NAD involving simple repeating sequence interspersed with random items. Stadler (1989) may have also revealed a form of visual NAD learning when he presented four adults with sequential search tasks, in which the final search could be predicted based on four of the six previous searches. In this task, the location of a target in the trials 1, 3, 4, and 6 made particular patterns that predicted where the target would be on trial 7. There were 24 specific patterns that predicted one of 4 outcomes on trial 7. Training was extremely extensive with 17 days of training and testing on 2688 trials per day, but on trials where the patterns were violated, participants’ responses were slower. Thus, it appears that visual-motor NAD learning is also possible under certain favorable conditions.

From a computational perspective, Onnis et al. (2015) made the point that the behavioral findings on NAD learning present challenges to virtually all current associative computational models. This is because associative learning models capitalize on the learning of local transition probabilities between successive elements, which is not sufficient to learn an NAD that spans across multiple intervening items. Onnis et al. (2015) compared predictions from simulations based on associative mechanisms [e.g., fragmentary knowledge or chunk strength measures, (Perruchet and Pacteau, 1990)] to those from connectionist models such as the simple recurrent network model (Elman, 1990). They found that not only could the connectionist models learn NADs (using input similar to that used by Gómez, 2002; Onnis et al., 2003 that manipulated the variability of the middle item), but also that they best captured the human data relative to the chunk-based models. Despite being a type of associative learning model itself, the simple recurrent network is computationally powerful because of its hidden layer units that represent temporal context, allowing it to form overlapping, graded representations that enable the learning not only of local AD transitions but, importantly, of NAD as well.

Despite the relative difficulty in showing learning of NAD by humans in the laboratory, learning of both AD (such as “rab” predicting “bit” in the word “rabbit”) and NAD (such as “he” predicting “-s” at the end of “walk” in “he walks”) appears to occur with relative ease in natural language learning, although the learning of NAD is likely only possible later in development when endogenous attentional mechanisms become available (de Diego-Balaguer et al., 2016). In natural language, both AD and NAD occur simultaneously in the same sequences or sentences and can even be made up of similar but separate stimuli (i.e., the same letters or words sometimes comprising AD and sometimes comprising NAD). For instance, any given word in a sentence consists of AD in the form of robust transitional probabilities between syllables; and yet these words themselves are often situated in the middle of other words and syllables that form an NAD. An example is: “He is positioning himself in the room.” The root word “position” consists of syllables that conform to adjacent dependencies, with higher transitional probabilities within the word than across word boundaries. This root word itself is straddled by the auxiliary verb “is” and the suffix “ing,” a non-adjacent dependency that allows for the grammatically correct inflection of “position”. Thus, both AD and NAD coexist in real-world, natural language situations.

Recently, both Romberg and Saffran (2013) and Vuong et al. (2016) showed that adults can learn AD and NAD in auditory sequences concurrently. Romberg and Saffran (2013) constructed artificial languages in which speech streams consisted of 3-word phrases with the first and third items having non-adjacent deterministic relationships and the intervening elements having adjacent probabilistic relationships with the other two items. There were 3 non-adjacent pairs in each language and 12 intervening items resulting in high intervening variability as in Gómez (2002). After listening to these phrases for 2–6 repetitions, participants were tested for familiarity of legal strings against foils in a 2AFC procedure, including confidence judgments. Adults were able to learn AD and NAD equally well within as little as 12 min of exposure (four repetitions) and, overall, showed improvements with increased exposure. However, although participants who were tested first on non-adjacent items performed equally well on adjacent and non-adjacent items and equally at all levels of exposure, those tested on adjacent items first performed better on adjacent items than non-adjacent and had increased performance after increased exposure for both types of items. This may suggest that, although both types of dependencies were learned at the same level, the learning of NAD was less robust. In accordance with Gómez (2002), highlighting the AD by testing them first made it more difficult to show learning of the NAD. In addition, higher confidence ratings on non-adjacent trials were associated with higher accuracy, while greater confidence on adjacent trials was not associated with greater accuracy. This may suggest that learning for the NAD was accompanied by explicit awareness while learning of AD was accomplished by more implicit means. In Romberg and Saffran’s (2013) second experiment, reducing the reliability of the adjacent items as predictors neither bolstered nor hindered learning of the NAD, contrary to Gómez’s (2002) finding that reducing the predictive nature of AD allowed participants to focus on information gained from NAD. Although these results appear to show equal levels of learning that are more fragile and explicit for NAD and more robust and implicit for AD, it is unclear how much of these findings stem from the adjacent and non-adjacent nature of the dependencies versus their deterministic and probabilistic nature. In addition, the relationships are further complicated by the fact that the two dependencies are not fully separated but intertwined in the stimuli. The AD is made up of items from the NAD paired with the intervening item. In other words, for the sequence ABC, A–C is the non-adjacent dependency and AB is the adjacent dependency, with the item A being part of both dependencies. Finally, although learning in this task is incidental and thus can potentially be implicit, learning is assessed in a way that requires participants to explicitly access their representations of the stimuli to make explicit judgments, which could be differentially influencing performance on the two types of dependency learning, as one or the other may be more or less accessible to conscious recall.

In another examination of AD and NAD learning, Vuong et al. (2016) tested adults’ ability to concurrently learn AD and NAD using a much lower level of variability in the intervening element. In addition, both types of dependencies were probabilistic with the same level of probability. Adjacent and non-adjacent dependencies were created from two separate sets of stimuli, pseudo words arranged in sequences of three. For non-adjacent sequences, the first word predicted the third word probabilistically and the intervening word was not predictive. For adjacent sequences, the first word predicted the second word probabilistically and the last word was not predictive. Training was accomplished through a modified serial reaction time (SRT) task, in which participants heard spoken versions of the sequences of pseudo words and responded by clicking on the printed form of each word on a computer as they heard the word. Thus, stimuli were auditory and responses were visuo-motoric. Training was extensive with 3 h-long training sessions distributed over successive days. Results indicated that with extensive training over multiple sessions, but without the methods used in other studies to highlight NAD, participants learned both types of dependencies. Learning was equivalent across dependency type in the online, implicit SRT measures but higher for the AD in offline prediction and explicit grammaticality judgment measures. Vuong et al. (2016) concluded that adults did not have difficulty learning AD and NAD simultaneously and learning of one type of dependency did not preclude learning of the other.

Studies of fully visual AD and NAD presented sequentially within the same sequence are much less abundant. One set of studies (Pacton and Perruchet, 2008; Pacton et al., 2015) presented a printed digit series that included both AD and NAD to participants in the context of a complicated cover task. The cover task consisted of searching for a target number, and each time it was found subtracting either the two digits immediately surrounding it (non-adjacent to each other) or immediately following it (adjacent to each other) depending upon the participant’s instructions. Then, they were asked to determine whether the difference was equal to 3. However, for most (but not all) of the experiments, digits were presented simultaneously rather than sequentially, although participants were told to read them left to right. When participants were subsequently given surprise two-alternative forced choice tests of AD and NAD, they found that adults could learn both visual AD and NAD. However, they could not learn both at the same time, and in fact only learned AD if the cover task emphasized the use of adjacent elements to complete the task and only learned NAD if the cover task emphasized the use of non-adjacent elements to complete the task. Thus, in this case, not only was highlighting of non-adjacent relationships necessary to learn NAD but highlighting of adjacent relationships was even necessary to learn AD. Pacton et al. (2015) found similar results using syllables as stimuli instead of digits. These findings seem contrary to Romberg and Saffran (2013) and Vuong et al.’s (2016) findings with auditory stimuli. It may be due to a difference between auditory and visual stimuli processing or perhaps due to the complicated nature of the cover task in Pacton and Perruchet (2008) and Pacton et al. (2015). Although they did include experiments to encourage deeper processing of all elements in the series by presenting them sequentially, which required keeping in mind one element while the following one or two items were presented, the working memory requirements of the cover task may have eclipsed the learning of relationships that were not highlighted, especially as it did not necessarily encourage patient’s memory of serial positions of all elements of the sequence.

Although it appears that adults can learn auditory AD and NAD concurrently, even without highlighting the NAD, as long as training is extensive (Vuong et al., 2016), a number of questions remain. First and foremost, to our knowledge, there have been no studies using visual stimuli presented sequentially (i.e., one stimulus at a time) with both AD and NAD present in all sequences (but for studies of NAD only that used simultaneously presented visual arrays rather than sequentially presented visual stimuli, see Sonnweber et al., 2015 for chimpanzees; and Barenholtz and Tarr, 2011, for humans). Given that concurrent learning of AD and NAD is possible with auditory stimuli (Romberg and Saffran, 2013; Vuong et al., 2016), investigating such learning in sequentially presented visual stimuli would be helpful for determining the extent to which such effects are robust across other perceptual domains. In addition, more work is needed to specify the cognitive processes involved in learning both types of dependencies. For instance, in the context of visual stimuli, does learning verbal adjacent and non-adjacent patterns rely on the same processes as does learning visual-spatial adjacent and non-adjacent patterns, or are there domain-specific constraints operating over such learning (e.g., Conway and Christiansen, 2006)? Likewise, are there different cognitive processes that support learning of AD versus NAD? It may be that some aspects of learning are more or less implicit than others and require more or less attention or working memory resources (Daltrozzo and Conway, 2014). For example, it is possible that learning NAD requires more attention and working memory resources than learning AD, as suggested by Romberg and Saffran (2013) and de Diego-Balaguer et al. (2016), though this proposal requires additional empirical support. In addition, more work is needed to understand to what extent each type of learning impacts different aspects of language ability.

It is also unclear to what extent the amount of exposure affects learning. Although Romberg and Saffran (2013) began to look at different amounts of exposure, even their longest exposure was relatively short and all at once (massed) rather than distributed across sessions, which typically results in better learning (Hovland, 1938). Vuong et al. (2016) provided longer, distributed training allowing for sleep consolidation but did not have a measure of how much learning may have increased from the addition of each session.

Finally, to date, most studies of NAD have used artificial word segmentation tasks with two alternative forced choice tests (Vuong et al., 2016, being a notable exception), a paradigm in which incidental learning takes place under presumably implicit circumstances, but in which participants are asked to make explicit judgments about test items. Using such an explicit measure of learning may underestimate participants’ knowledge, compared to an indirect measure of learning that does not require participants to explicitly access their knowledge (Redington and Chater, 1996). Furthermore, the vast majority of these studies have used only a single intervening element between items in a non-adjacent dependency pair (but see Remillard, 2008, for longer non-adjacent dependencies in an SRT task), and the effect of an increased number of intervening elements is largely unknown. In addition, the intervening items and NAD elements, as well as AD and NAD in studies that have included both, have often come from perceptually different item sets. Although this may be a desirable condition to elicit the best performance and determine whether NAD and AD learning is even possible, it is not ecologically consistent. As mentioned previously, AD and NAD in the natural world can often consist of highly related and intertwined stimuli in the same sequence, such as in spoken and written natural language.

To begin to address these critical questions, we first tested whether adults could learn AD and NAD within the same set of sequences presented in visuo-spatial and visuo-verbal formats during a single exposure and testing session (Experiment 1). We used a memory based reproduction task that encouraged deep processing of each element and its serial position in the sequence and in which learning of dependencies was incidental and therefore potentially implicit; testing was surreptitious and indirect so that responses did not require explicit access to knowledge. In addition, the AD pairs were used as the intervening elements between the NAD pairs such that there were always two items (two serially presented spatial locations or two serially presented syllables) between every NAD predictive pair and both AD and NAD were made of the same elements put into different dependency pairs. However, the AD and NAD themselves were independent of each other; that is, the particular occurrence of an AD had no bearing on or relationship to the specific instantiation of the NAD, and vice-versa. In a second experiment (Experiment 2), a subset of the same participants took part in a second session containing the same sequential learning tasks as before, in order to examine how learning of the two types of dependencies was affected by amount of exposure over time. Additionally, to obtain a better understanding of the cognitive processes most associated with AD and NAD learning, we also administered neuropsychological measures to determine the specific relationships between learning of AD and NAD and language and other core cognitive abilities.

We hypothesized that under these ecologically consistent circumstances that encouraged deep processing and allowed for both implicit learning and indirect testing, adults would be able to learn AD and NAD within the same set of sequences in both visuo-spatial and visuo-verbal formats within a single session. This finding would be consistent with the results of Romberg and Saffran (2013) and Vuong et al. (2016) with auditory stimuli. If, however, AD is learned more easily and effectively compared to NAD as previous research suggests, then AD should show a higher level of learning than NAD. This should also manifest itself in different patterns of learning across time for the two types of dependency; AD learning should reach a relatively high level of learning quickly and remain robust over time (e.g., Romberg and Saffran, 2013). On the other hand, the learning of NAD may be more fragile and susceptible to outside influences making it less stable over time (e.g., Romberg and Saffran, 2013). Finally, the investigation into relationships between AD and NAD learning and various cognitive and language measures is exploratory, but some tentative predictions are made here. One possibility is that performance on visuo-spatial sequential learning will be positively correlated with a spatial span measure, and performance on visuo-verbal sequential learning will be positively correlated with verbal span. However, it is also possible that there could be interference between the processes underlying the learning of dependencies in different domains (e.g., Kirby, 1993). In such a case, we might expect visuo-spatial sequential learning to be negatively correlated with verbal span and visuo-verbal sequential learning to be negatively correlated with spatial span. Other measures that may be related to AD and NAD learning include attention and inhibition (measured here by Eriksen’s Flanker task, Eriksen and Eriksen, 1974), processing speed (measured here by the Symbol Digit Modality Test, Smith, 1982), and language (measured here by the Sentence Completion subtest of the Comprehensive Assessment of Spoken Language, Carrow-Woolfolk, 1999).

## Experiment 1

### Materials and Methods

#### Participants

Participants were 59 adults (36 female) aged 18–29 (*M* = 21), recruited from Georgia State University and the surrounding Atlanta area (both students and non-students). All were native English speakers and reported no known cognitive, motor, language, hearing or uncorrected vision impairments. All except one were right handed (a criterion to participate in a later fMRI study, the results of which are not reported in the current manuscript). Students enrolled in a course requiring experiment credit were given 3 credits. All others were paid $25 for participation. Fourteen additional participants were excluded from analyses: 4 due to computer malfunctions, 5 due to failure to follow instructions resulting in data not being recorded, 4 due to discontinuation by the participant before conclusion of the experiment session, and one was later found to have brain lesions in an incidental MRI finding. All participants gave written informed consent in accordance with the Declaration of Helsinki. All procedures were approved by the Center for Advanced Brain Imaging institutional review board.

#### Materials

**FIGURE 1:**
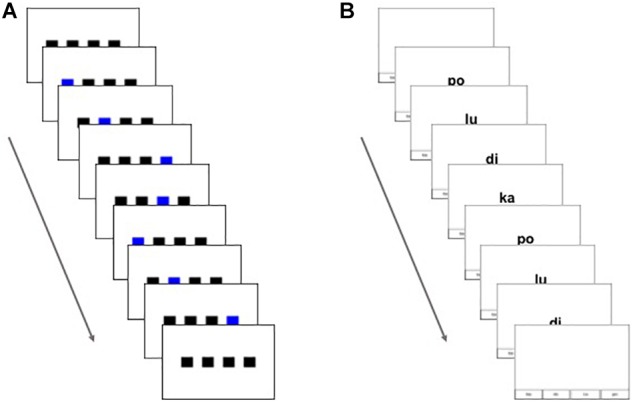
Figure shows presentation over time of a single trial of **(A)** the visuo-spatial sequential learning task and **(B)** the visuo-verbal sequential learning task.

We designed two types of sequential learning tasks: visuo-spatial and visuo-verbal. The visuo-spatial task consisted of black squares that flashed sequentially on a computer screen, similar to previous artificial grammar learning tasks used, for example, in Karpicke and Pisoni (2004) and Conway et al. (2010) (see Figure 1A). For this task, four black squares were aligned horizontally in the center of the screen. All squares remained on the screen throughout sequence presentation on each trial. Sequences were indicated by squares individually flashing blue for 400 ms at their turn within the sequence. The blue moved continuously from one sequential position to the next with an inter-stimulus interval (ISI) of 200 ms. This ISI was short enough that the subjective experience of the participant was expected to be that of continuous motion from one position to the next with no pause in between. When the sequence presentation on a given trial was complete, a blank white screen was presented for 500 ms. The row of black squares then reappeared on the screen, which was the participant’s cue to reproduce the sequence using the 1 through 4 keys on a standard computer keyboard to indicate each of the four locations. As the participant pressed the buttons, the corresponding square flashed blue. If the participant pressed at least one key in response, the program waited for 3 s after each press to allow for further responses. When there was no further response within 3 s of the last key press, the next sequence was presented. If the participant did not respond within 13 s, the next sequence was presented. On the surface, this task may seem similar to a serial reaction time (SRT) task, but the cognitive demands and response requirements are different. In the SRT task, stimulus items are presented sequentially, and the participant responds as quickly as possible to each item separately as it is displayed by pressing a button corresponding to the item displayed. The measure of interest is response time. The task described here is a sequence reproduction task in which an entire sequence of items is presented to the participant, and upon completion of the sequence, the participant is told to reproduce the same sequence as accurately as possible from memory (with no mention of what the rate of response should be). The measure of learning of dependencies is the accuracy of recall of the entire sequence (Karpicke and Pisoni, 2004; Conway et al., 2010). This measure of learning is similar to that used in the Hebb repetition effect, which also uses serial recall accuracy to assess learning of patterns presented in input sequences (e.g., Page and Norris, 2009). Like the SRT task, our task does not explicitly draw attention to patterns, dependencies, or rules; but unlike the SRT, it requires participants to recall each sequence in its entirety before responding, likely involving a deeper level of processing of the sequence as a whole.

The visuo-verbal task consisted of printed nonsense syllables that were presented sequentially on the screen (see Figure 1B). Four orthographic nonsense syllables were used to construct the sequences: ka, po, lu, and di. Syllables appeared individually in the center of the screen to form the sequences. Throughout sequence presentation and response, a representation of the location of the response button (keyboard 1 through 4 as in the spatial task) for each syllable remained at the bottom of the screen to simplify the participant’s task of mapping the syllables to response buttons while reproducing the sequences (see Figure 1B), as well as to make the task more similar to the visuo-spatial task in which the squares remained on the screen throughout sequence presentation and response. Although this mapping between syllables and buttons gave the response a visuo-spatial component, the input was fully visuo-verbal. Individual syllables were presented for 400 ms continuously with an ISI of 200 ms. When the sequence presentation of a trial was complete, a blank white screen was shown for 500 ms. Again, the cue for the participant to respond was the reappearance of the syllable mapping at the bottom of the screen. The participant used the 1–4 keys on a standard keyboard to reproduce the visuo-verbal sequence. As the participant pressed the buttons, the corresponding syllables were shown on the screen. Again, participants had 13 s to begin responding and 3 s for each button press.

##### Artificial grammar

For both tasks, sequences conformed to an artificial grammar that dictated both AD and NAD within each sequence. For each task (spatial and verbal), four pairs of non-adjacent dependencies and four pairs of adjacent dependencies were created (see Table 1). Thus, AD and NAD were composed of the same items (but following different rules) so as not to artificially highlight either type of dependency or to make the different types of dependencies more salient by using different types of stimuli for each. We purposefully designed the task this way, in order to be more similar to sequential dependencies found in real-world domains, such as language. Such a design was expected to make learning quite difficult, given that previous studies have generally found that NAD were unlikely to be learned in auditory sequences unless they were highlighted by, for example, perceptual differences between AD and NAD stimuli (e.g., Creel et al., 2004). Success at learning both AD and NAD in this study, therefore, would represent a novel and particularly impressive finding.

**Table 1:** List of adjacent and non-adjacent pair rules.

Adjacent pairs	Non-adjacent pairs^a^
A–D	A-×-B
B–A	B-×-C
C–B	C-×-D
D–C	D-×-A


*Both Adjacent and non-adjacent pairs were made up of the same items (A, B, C, and D). For the spatial task, A, left most square; B, second from the left; C, second from the right; D, right most square. For the verbal task A, ka; B, po; C, lu; and D, di. ^*a*^The intervening element in the non-adjacent pairs (×) consisted of any of the adjacent pairs and therefore consisted of two items*.

**FIGURE 2:**
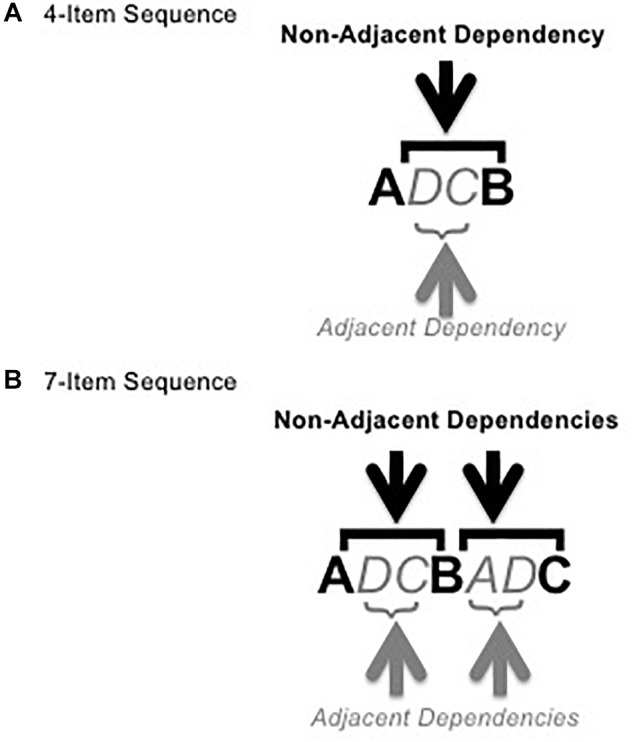
Figure shows example 4-item **(A)** and 7-item **(B)** grammatical sequences with adjacent and non-adjacent pairs marked (1 each in the 4-item sequence, 2 each in the 7-item sequence). Bold, italics, and color are for illustrative purposes only.

To form sequences, the adjacent pairs were used as the intervening element (x) between the non-adjacent pair items (see Figure 2). Each sequence was either composed of a single non-adjacent dependency pair and a single adjacent dependency pair (4 total items, for exposure to pairs only, see Figure 2A) or two of each type of pair with the second item of the first non-adjacent pair becoming the first item of the second non-adjacent pair (7 total items, for exposure and test, see Figure 2B). All dependencies were deterministic. Thus, in all sequences, the first item of an adjacent or non-adjacent pair, regardless of position in the sequence, was always 100% predictive of the second item in the pair. These made up the grammatical sequences, 16 4-item sequences and 64 7-item sequences (32 for exposure and 32 for test). See Appendix A for a list of grammatical test sequences.

The 32 7-item grammatical test sequences were also used to create 32 7-item ungrammatical sequences. Half of the grammatical sequences had violations introduced into both adjacent pairs by replacing one member of each pair with an incorrect item. This created 16 “adjacent ungrammatical sequences.” The other half of the grammatical 7-item sequences were given similar violations in both non-adjacent pairs to make 16 “non-adjacent ungrammatical sequences.” See Appendix A for a list of ungrammatical test sequences. Although all of the grammatical sequences were made with the same adjacent and non-adjacent pairs and thus did not differ in adjacency, we will call those grammatical sequences used to make the adjacent ungrammatical sequences “adjacent grammatical” and those used to create the non-adjacent ungrammatical sequences “non-adjacent grammatical”. We will use adjacent grammatical sequences for comparison with adjacent ungrammatical sequences and non-adjacent grammatical sequences for comparison with non-adjacent ungrammatical sequences, since each grammatical sequence only differs from its ungrammatical pair by the 2 violations. See Appendix A for further explanation.^1^

To avoid frequency effects, each item (represented by letters A through D in Appendix A, Table 1, and Figure 2) was located in each sequence position (1st through 7th) exactly 4 times for each type of sequence (grammatical adjacent, grammatical non-adjacent, ungrammatical adjacent, and ungrammatical non-adjacent).

##### Cognitive assessments

We used several standardized measures as well as one well-known non-standardized task to obtain cognitive performance data. Sequential learning has been hypothesized to be closely related to a number of cognitive skills. These skills include: language (e.g., Saffran et al., 1996); attention span and working memory, which have been suggested as potentially being required for more explicit but not implicit forms of learning (e.g., Daltrozzo and Conway, 2014) and may be particularly important for holding NAD in mind across intervening items (e.g., Romberg and Saffran, 2013; de Diego-Balaguer et al., 2016); selective attention and inhibition, which likewise may be beneficial for noticing and attending to NAD relationships (e.g., de Diego-Balaguer et al., 2016); and processing speed, which may be important for processing items presented sequentially at a high rate of speed. Thus, we measured each of these skills to conduct exploratory analyses to determine which cognitive variables are associated with AD and NAD learning. We also measured intelligence to rule out IQ or broad intelligence as an underlying factor accounting for any relationships between cognitive measures and sequential learning.

###### Language

We assessed language ability with the Sentence Completion subtest of the Comprehensive Assessment of Spoken Language (CASL, Carrow-Woolfolk, 1999). In this standardized test, the experimenter reads sentence stems of increasing length, complexity, and vocabulary level with the final word left out. The participant is asked to provide a single word, of which no form has been used in the sentence, to complete the sentence semantically and make it grammatically correct. The assessment is not timed. This assessment was chosen because previous research has suggested that making predictions about upcoming linguistic units during spoken sentence processing is associated with statistical-sequential learning ability (e.g.,Conway et al., 2010).

###### Attention span and working memory

Auditory-verbal attention span and working memory were assessed with the Digit Span Forward and Digit Span Backward of the Wechsler Adult Intelligence Scales – Fourth Edition (WAIS-IV, Wechsler, 2008). In the Digit Span Forward, the experimenter reads out sequences of digits increasing in length. The participant immediately verbally repeats the sequence in the same order that it was presented. For the Digit Span Backward, the experimenter reads different digit sequences of increasing length, and the participant repeats each sequence in the reverse order to which it was given by the experimenter. The Digit Span Forward provides a measure of verbal attention span (e.g., Kieffer-Renaux et al., 2000), and the Digit Span Backward is a measure of verbal working memory.

Visuo-spatial attention span and working memory were assessed with the Spatial Span Forward and Spatial Span Backward of the Wechsler Adult Intelligence Scales – Revised (WAIS-R, Wechsler, 1991). The procedure is the same as for the Digit Span Forward and Backward, except that instead of hearing digits and responding verbally, the participant watches the experimenter touch sequences of cubes in particular locations on a board, and then responds by touching them in the same or reversed order. The Spatial Span Forward is a measure of visuo-spatial attention span and the Spatial Span Backward of visuo-spatial working memory.

###### Selective attention and inhibition

Selective attention and inhibition were measured with a computerized version of the Eriksen Flanker Task. In this task, participants are presented with horizontally arrayed arrows and their task is to indicate which direction the center arrow is facing using the computer keyboard. The arrows flanking the center arrow may be facing the same direction as the center (congruent, →→→→→) or the opposite direction (incongruent, →→←→→). Response times are generally slower for incongruent trials. Larger average differences between congruent and incongruent trials have been interpreted in the literature to reflect poorer selective attention and inhibition (Eriksen and Eriksen, 1974). We used the Salthouse (2010) composite score method that takes into account both response time and accuracy and removes outlier trials.

###### Processing speed

Processing speed was measured with both the written and oral versions of the Symbol Digit Modality Test (Smith, 1982). This is an assessment in which participants are given a key matching various visual symbols to the digits 1 through 9. After a brief practice, participants are given the written test in which they have 90 s to write as many of the corresponding numbers below each symbol in a list as they can. The oral version is the same except that the participant states the numbers verbally instead of writing each down.

###### Intelligence

IQ was measured with the Wechsler Abbreviated Scales of Intelligence – Second Edition (WASI-II, Wechsler and Zhou, 2011) Two-Subtest Form. The Two-Subtest form consists of the Vocabulary and Matrix Reasoning subtests which together provide an estimate of IQ. The Vocabulary subtest measures expressive vocabulary and verbal concept formation. Matrix Reasoning requires the participant to solve visual pattern completions and analogies among a matrix of visual-spatial patterns with one item in the matrix missing. The participant must choose the missing item from a multiple-choice array. Matrix Reasoning subtest requires visual-spatial problem-solving and concept formation.

#### Procedure

All participants followed the same procedure and completed the same tasks and assessments in the same order. Although presentation of sequences within each task was randomized, task order was not counterbalanced. The decision not to counterbalance was made because it was intended that a subset of the participants would subsequently participate in a separate fMRI study, in which participation in this study would serve as exposure to the sequences used in the fMRI study. For that study, we wished to remove as much variability in brain activation as possible due to such extraneous variables as order of task exposure. Correspondence between spatial location or syllable and sequence element (as represented by A, B, C, and D in the sequence list of Appendix A) was randomized for each task, for each participant to minimize the likelihood of interference or facilitation from one sequential learning task to the next. Order of task presentation for the current study was as follows:

(1) Visuo-Spatial Sequential Learning Task
(2) Cognitive Assessments
(3) Visuo-Verbal Sequential Learning Task

##### Visuo-spatial sequential learning task

After informed consent was completed, participants were led to a private, sound-attenuated room and seated in front of a Dell Optiplex 990 personal computer running Windows 7 Enterprise with a standard keyboard and a 17 inch ELO touchscreen monitor. An introduction to the visuo-spatial task was given verbally by the experimenter and the task was presented with Eprime 2.0 psychology experiment presentation software. The entire task took approximately 20 min.

###### Mapping

The experimenter explained to the participant that the purpose of the mapping portion of the task was to learn which keyboard buttons (numbers 1 through 4) were associated with which squares on the screen when responding. The participant was asked to move the keyboard to the most comfortable position to respond on the 1 through 4 keys, without accidentally hitting other keys using only their right hand. The “1” key corresponded to the left-most square, and the “2” key corresponded to the next key to the right, etc. The participant was given verbal as well as on-screen printed instructions to press the corresponding key each time one of the black squares turned blue. The mapping training was then started. Participants were presented 16 randomly ordered trials, with a single square turning blue on each trial, 4 trials for each of the 4 locations. On each trial, the participant received accuracy feedback and was required to correct an incorrect response before moving on. The experimenter stayed with the participant and monitored the task to ensure that the participant understood the task and completed it with at least 80% accuracy on first responses. All participants reached 80% accuracy.

###### Exposure: 4-item sequences

Once the mapping section was over, participants were given a chance to ask questions. They were then told that they would now be replicating sequences, first a set of 4-item sequences followed by a set of 7-item sequences. They were reminded to use only their right hand to respond and keep the keyboard in a comfortable position. Participants were not given any instruction on whether or not to respond quickly. From anecdotal self-report and observation of pilot participants, it appeared that many participants followed the same rhythm of stimulus presentation for their responses, regardless of difficulty, while others responded as quickly as they could, regardless of difficulty, and others’ responses were more variable. This bimodal strategy for responding renders response time unreliable as a measure of performance, and thus accuracy was used instead. The experimenter then started the rest of the experiment (including the two exposure phases and the test phase, which were not labeled as such for participants), and left the room. This first exposure phase was meant only to help participants learn the adjacent and non-adjacent pairs under simpler learning conditions; each sequence included only one adjacent and one non-adjacent pair for a total of four items per sequence (see Figure 2A). Participants were simply told that they would see sequences of four images (squares) and that after a sequence was presented, they were to reproduce the sequence in the same order by pressing the correct keys as they had learned in the mapping phase. Participants completed 32 4-item trials, composed of 2 presentations, each of 16 sequences in random order. All sequences followed the grammar by always containing a single “legal” adjacent pair and a single “legal” non-adjacent pair in the prescribed format. Participants were not told that there was an underlying grammar or that there were AD and NAD embedded in the sequences. Performance on 4-item was not analyzed as all sequences were grammatical and did not give any information on learning of the underlying grammar.

###### Exposure: 7-item sequences

Upon completion of the 4-item set of sequences, participants took a self-paced break during which they read instructions telling them that in the next section they would see 7-item sequences and that they should replicate them in the same way as in the previous section. When the participant pressed a key to start the next session, he or she was presented with 64 trials of grammatical 7-item sequences, two each of 32 sequences that consisted of two “legal” adjacent pairs and two “legal” non-adjacent pairs (see Figure 2B). Sequences were presented in random order. As before, participants were not instructed about the nature of the embedded dependencies.

###### Test

When the exposure phases were complete, participants took another self-paced break during which they read instructions that were exactly the same as those for the exposure sections. For this test section, they were randomly presented with 128 trials (two each of 64 sequences). All of the sequences were new to the participants, with half of them following the same grammar as presented during exposure and the other half containing grammar violations as described previously. Half of the ungrammatical sequences were adjacent ungrammatical, meaning that they had an incorrect item within each adjacent pair. The other half were non-adjacent ungrammatical with incorrect items in both non-adjacent pairs. Participants were not given any indication that this section served as a test or that some sequences contained sequential violations. Accuracy data were collected.

##### Cognitive assessments

Following the first sequence learning task, the participant was led to a separate quiet room, where a trained tester sat across the table from the participant and presented the standardized assessments in the following order: Sentence Completion, Digit Span Forward, Digit Span Backward, Spatial Span Forward, Spatial Span Backward, Vocabulary, Matrix Reasoning, written Symbol Digit Modality Test, and oral Symbol Digit Modality Test. Once the standardized assessments were completed, the experimenter took the participant back to the original room and instructed him or her to complete the Eriksen Flanker task presented with Eprime 2.0 on a 12.5 inch Lenovo Thinkpad laptop and notify the experimenter when it was completed.

##### Visuo-verbal sequential learning task

For the visuo-verbal task, the participant was returned to the same Dell personal computer that was used for the visuo-spatial learning task. Like the first learning task, the verbal task also included a brief mapping training to familiarize the participant with which key on the keyboard (numbers 1 through 4) was associated with which syllable. Participants viewed single syllables appearing on the screen one at a time with the participant responding by pressing the corresponding key with corrective feedback. The syllables, ka, po, lu, and di, were printed at the bottom of the screen to remind the participant that the 1 key went with ka, 2 with po, 3 with lu, and 4 with di. That mapping reminder remained on the screen throughout the mapping training as well as during sequence presentation and response during the exposure and test phases. The rest of the mapping section, as well as the exposure and test phases, followed the exact some procedure and trial count as the visuo-spatial task (except that the sequences were composed of syllables). It also took approximately 20 min to complete.

Importantly, the mapping between the elements of the grammar (“A,” “B”, etc.) and the specific non-word tokens (“ka,” “po,” etc.) or spatial locations was randomly determined for each participant. Thus, although the two sequential learning tasks involved the same underlying grammar, both the input stimuli and the specific motoric responses were different across the tasks, and thus, interference or facilitation between the two tasks was expected to be minimal.

### Results

#### Learning

Previous studies have shown that if participants learned the regularities embedded in the sequences, their ability to reproduce sequences following the grammar should be better than their ability to reproduce sequences containing violations of the grammar (Karpicke and Pisoni, 2004; Conway et al., 2010). To test for learning, paired samples *t*-tests were performed to compare the total number of items reproduced correctly (correct item in the correct serial position) out of 224 (7 items per sequence times 32 sequences) for grammatical versus ungrammatical sequences. Separate *t*-tests were done (four in all) for sequences with adjacent and sequences with non-adjacent violations within each of the tasks, visuo-spatial and visuo-verbal. Requiring the correct items to be reproduced in the correct order was a relatively strict measure of learning, requiring recall of the sequence in its entirety, including both the adjacent and non-adjacent structure. Measuring the total number of items correct across sequences of the same type provided a more sensitive measure of learning with a greater range than the mean number of correct items per sequence or the number of error-free sequences.

**FIGURE 3:**
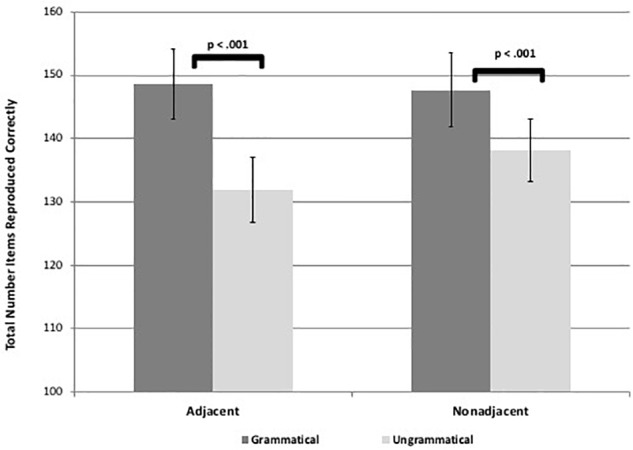
Figure shows visuo-spatial task learning as demonstrated by significant differences in total number of items reproduced between grammatical and ungrammatical sequences. Error bars represent standard errors.

**FIGURE 4:**
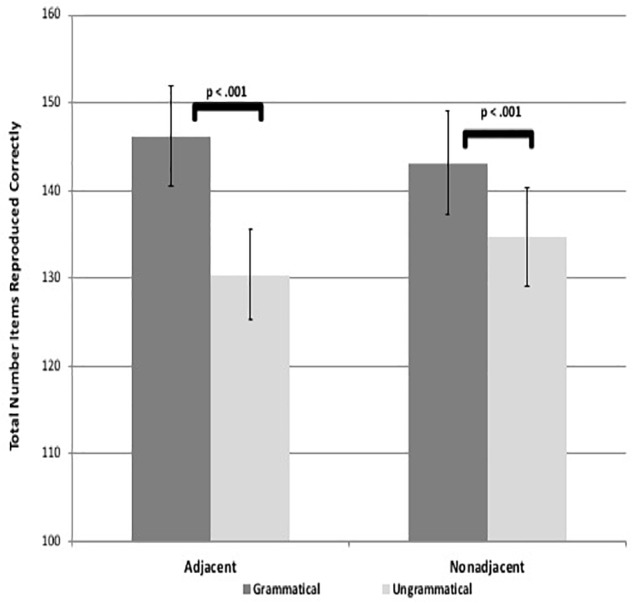
Figure shows visuo-verbal task learning as demonstrated by significant differences in total number of items reproduced between grammatical and ungrammatical sequences. Error bars represent standard errors.

Results revealed that participants reproduced significantly more items for the grammatical than ungrammatical sequences for both AD and NAD sequences in both visuo-spatial and visuo-verbal formats, suggesting they learned both types of dependencies for both tasks [spatial adjacent: *t*(58) = 7.47, *p* < 0.001, *d* = 1.00, spatial non-adjacent: *t*(58) = 4.70, *p* < 0.001, *d* = 0.61, verbal adjacent: *t*(58) = 7.50, *p* < 0.001, *d* = 0.97, verbal non-adjacent: *t*(58) = 3.92, *p* < 0.001, *d* = 0.51; see Figure 3, 4]. These results suggest that adults are capable of learning adjacent and non-adjacent dependencies concurrently in both visuo-spatial and visuo-verbal sequences, with only a single, brief exposure session, even when non-adjacent dependencies are not highlighted.

Because there were more repetitions in the grammatical than in the ungrammatical sequences, performance on the grammatical sequences could have simply been enhanced by the facilitating effect of repetitions on recall. However, when independent *t*-tests were conducted using only sequences without repetitions (and using mean number correct per sequence as the dependent variable since there were unequal numbers of total items per condition), results were the same (see Table 2). The full set of sequences, regardless of repetition status, was used for all further analyses.

**Table 2:** Results of paired samples *t*-tests between grammatical and ungrammatical sequences on mean number of correct items per sequence using only sequences without repetitions.

	Grammatical		Ungrammatical					
						
Task and dependency	*M*	*SD*	*M*	*SD*	*n*	*t*	*p*	*d*
Spatial adjacent	5.11	1.57	3.84	1.32	59	11.14	<0.001	1.45
Spatial non-adjacent	4.70	1.67	3.95	1.20	59	5.20	<0.001	0.68
Verbal adjacent	5.03	1.51	3.96	1.26	59	10.44	<0.001	1.35
Verbal non-adjacent	4.82	1.72	4.00	1.28	59	6.37	<0.001	0.83


In addition, because analyses were done on a total number of items correct in each type of sequence and because there were both adjacent and non-adjacent pairs in all sequences, it is possible that the learning effect was carried by adjacent pairs in both AD and NAD sequences. This would mean that only AD pairs were learned but that violations in either AD or NAD pairs in the ungrammatical sequences caused the number of AD pair items reproduced on ungrammatical trials of either type to be reduced. Although it seems unlikely that a violation of an unlearned NAD pair would caused disruption of AD recall (but violation of a *learned* NAD pair could cause disruption to reproduction of the whole sequence including both types of dependency), *t*-tests were done separately for items that made up adjacent pairs and items that made up non-adjacent pairs. Note that although there were equal numbers of AD and NAD pairs, there were more AD items than NAD items because one NAD item was shared as the second item of one pair and first item of the other pair in each sequence. For AD, there were 4 items per sequence for a total of 128 items forming 64 pairs (2 per sequence). For NAD, there were 3 items per sequence for a total of 96 items forming 64 pairs (2 per sequence). In addition, sample sizes were slightly smaller because files from one participant for the spatial task and two participants for the verbal task contained only the total numbers correct, not where errors were made within each sequence due to computer program malfunction. Results for both adjacent and non-adjacent items indicated a learning effect in all types of sequences (see Table 3).

**Table 3:** Results of paired samples *t*-tests between grammatical and ungrammatical sequences on total number of adjacent items correct or total number of non-adjacent items correct per sequence for each type of sequence.

		Grammatical		Ungrammatical					
							
Type of dependency	Type of sequence	*M*	*SD*	*M*	*SD*	*n*	*t*	*p*	*d*
Adjacent dependency	Spatial adjacent	82.81 (64.70%)	24.27	72.00 (56.25%)	22.78	58	7.13	<0.001	0.93
	Spatial non-adjacent	82.31 (64.30%)	25.70	76.33 (59.63%)	22.08	58	4.55	<0.001	0.58
	Verbal adjacent	81.46 (63.64%)	23.81	71.40 (55.78%)	22.40	57	6.94	<0.001	0.92
	Verbal non-adjacent	78.68 (61.47%)	24.32	74.79 (58.43%)	24.09	57	2.80	0.007	0.38
Non-adjacent dependency	Spatial adjacent	66.47 (69.24%)	18.22	59.52 (62.00%)	18.03	58	7.31	<0.001	0.96
	Spatial non-adjacent	66.60 (69.38%)	19.47	62.66 (65.27%)	16.91	58	4.41	<0.001	0.54
	Verbal adjacent	66.07 (68.82%)	19.57	59.70 (62.19%)	17.52	57	6.89	<0.001	0.95
	Verbal non-adjacent	65.88 (68.63%)	19.27	60.96 (63.5%)	18.48	57	4.76	<0.001	0.65


In summary, significant learning was observed for both AD and NAD patterns in both visuo-spatial and visuo-verbal formats.

#### Effects of Adjacency and Task

To examine learning differences among conditions, we constructed percentage change scores. Although difference scores are often used as a measure of learning (for example, for this study, one difference score would be the total adjacent grammatical items correct minus total adjacent ungrammatical items correct), difference scores do not take into account the baseline level of performance, such that a participant who scored 25 grammatical correct and 15 ungrammatical correct would have the same difference score of 10 as someone who got 55 and 45 correct. However, the percentage change from grammatical to ungrammatical in the first example is 40%, while in the second example it is only 18%, suggesting that although the first participant reproduced fewer items correctly overall, he or she evidenced a greater magnitude of learning. Thus, we used percentage change scores as a measure of learning that takes into account baseline level of performance.

We calculated percent change for each condition as

$$\left(\frac{\left(U-G\right)}{G}\times 100\right)$$

in which *G* is the total number of grammatical items correct and *U* is the total number of ungrammatical items correct. This represents the extent to which performance was facilitated for grammatical sequences compared to ungrammatical sequences, relative to baseline performance on grammatical sequences. Because lower performance on ungrammatical sequences than grammatical sequences indicates learning, the percentage change formula would give lower (more negative) scores for higher amounts of learning, which seems unintuitive. Thus, to make higher (more positive) scores represent more learning, we multiplied all scores by -1.

**FIGURE 5:**
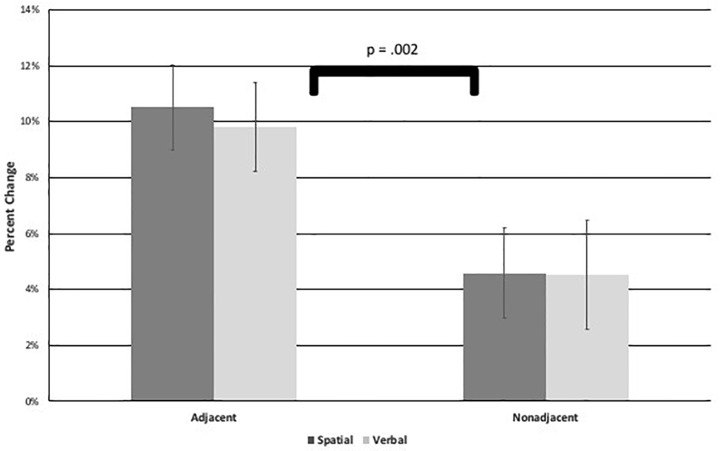
Figure shows the Experiment 1 task × adjacency Analysis of Variance on percent change scores results with a significant main effect of adjacency. Error bars represent standard errors.

To compare the level of learning between conditions, a 2 (task: spatial or verbal) × 2 (adjacency: adjacent or non-adjacent) repeated measures ANOVA was performed on percentage change scores. Results indicated the only significant effect to be a main effect of adjacency, *F*(1,58) = 10.10, *p* = 0.002, η^2^ = 0.15 (see Figure 5), in which participants showed a higher percentage change, and thus, greater learning for adjacent dependencies (*M* = 10.17%, *SD* = 12.06) than non-adjacent dependencies (*M* = 4.55%, *SD* = 13.75). There was no difference between spatial and verbal tasks. Therefore, AD was learned to a greater degree than NAD regardless of whether they were presented in visuo-spatial or visuo-verbal sequences. In addition, learning appeared robust in that the majority of participants evidenced positive percentage change, especially in the adjacent conditions: 86% of participants had positive percentage change of spatial AD sequences, 80% for spatial NAD, 90% for verbal AD, and 68% for verbal NAD.

Because 20 out of the total of 64 violations in the NAD sequences presented (involving 9 out of the 16 sequences) made new AD pairs (albeit in the wrong locations), it is possible that some ungrammatical NAD sequences became more reproduceable because they contained more AD pairs. This would have the effect of increasing the ungrammatical NAD total correct and decreasing the difference between grammatical and ungrammatical NAD, and thus, percentage change. This might make learning of NAD look lower than it actually was. Therefore, the 9 ungrammatical NAD sequences containing these new AD pairs were removed from NAD percentage change scores, along with their 9 counterpart grammatical NAD sequences, and the above repeated measures ANOVA was recalculated with the same results, only a main effect of adjacency: *F*(1,58) = 10.58, *p* = 0.002, η^2^ = 0.89; AD *M* = 10.17%, *SD* = 12.06; NAD *M* = 4.24%, *SD* = 22.52. The full set of sequences was used for further analyses.

Although the task was not designed for response times as a measure of learning, we conducted an analysis of percentage change in median latency (milliseconds) with a 2 (task: spatial or verbal) × 2 (adjacency: adjacent or non-adjacent) repeated measures ANOVA. Results indicated a main effect of adjacency, with a greater percentage change for NAD relative to AD sequences: *F*(1,56) = 4.73, *p* = 0.035, η^2^ = 0.09; AD *M* = 3.81%, *SD* = 17.53; NAD *M* = 9.39%, *SD* = 19.96. The analyses of the RT data suggest that the learning effect was greater for NAD than AD, despite the accuracy scores showing the opposite effect. However, we believe that the RT data should be interpreted with caution due to the large amount of variability in the responses (as indicated by the standard deviations) and that the task (and instructions) were not designed to encourage participants to respond quickly, but rather to respond accurately.

#### Correlations With Cognitive Assessments

To determine the relationships between learning of AD and NAD (using percentage change scores) and various cognitive measures, specifically language, working memory, attention, and processing speed, correlation analyses were conducted. We used partial correlations controlling for IQ to ensure that any correlations between adjacent and non-adjacent sequential learning and cognitive assessments were not accounted for by general intelligence. Due to the exploratory nature of the correlational analyses, p-values were not adjusted for multiple comparisons, and thus, strong caution is urged in the interpretation of results. Cognitive assessment data for two participants were not complete due to experimenter error, thus those participants were excluded from correlational analyses leaving a sample size of 57.

Significant partial correlations with sequence task percentage change scores controlling for IQ were as follows (see Table 4 for full correlation matrix). Visuo-spatial AD percentage change learning score was significantly positively correlated with Eriksen’s Flanker interference score, *r*(54) = 0.484, *p* < 0.0001. Visuo-spatial NAD percentage change learning score was significantly negatively correlated with Forward Digit Span *z*-score, *r*(54) = -0.293, *p* = 0.029, and Backward Digit Span *z*-score, *r*(54) = -0.335, *p* = 0.012. Visuo-verbal NAD percentage change learning score was significantly positively correlated with Sentence Completion standard score, *r*(54) = 0.285, *p* = 0.033 and significantly negatively correlated with Forward Spatial Span *z*-zcore, *r*(54) = -0.268, *p* = 0.046.

**Table 4:** Partial Correlations between Experiment 1 sequential learning measures and cognitive measures controlling for IQ (*n* = 57).

Variables		1	2	3	4	5	6	7	8	9	10	11
(1) Spatial adjacent percent change	*r*	1.000										
	*p*-value											
(2) Spatial non-adjacent percent change	*r*	-0.007	1.000									
	*p*-value	0.956										
(3) Verbal adjacent percent change	*r*	0.082	-0.069	1.000								
	*p*-value	0.546	0.612									
(4) Verbal non-adjacent percent change	*r*	-0.157	0.105	-0.060	1.000							
	*p*-value	0.248	0.439	0.660								
(5) Forward digit span	*r*	0.043	-0.293	-0.068	-0.219	1.000						
	*p*-value	0.751	0.029	0.618	0.105							
(6) Backward digit span	*r*	0.071	-0.335	-0.191	0.023	0.392	1.000					
	*p*-value	0.601	0.012	0.158	0.868	0.003						
(7) Forward spatial span	*r*	0.027	-0.068	-0.139	-0.268	0.119	0.252	1.000				
	*p*-value	0.844	0.618	0.307	0.046	0.381	0.061					
(8) Backward spatial span	*r*	0.019	0.038	-0.020	0.003	-0.117	-0.071	0.273	1.000			
	*p*-value	0.890	0.779	0.883	0.983	0.392	0.601	0.042				
(9) Flanker	*r*	0.484	-0.007	0.186	-0.038	0.216	-0.012	0.100	0.204	1.000		
	*p*-value	0.000	0.958	0.169	0.780	0.110	0.928	0.462	0.132			
(10) Sentence completion	*r*	-0.002	0.067	-0.039	0.285	0.010	-0.032	0.085	-0.014	-0.055	1.000	
	*p*-value	0.990	0.622	0.773	0.033	0.944	0.815	0.533	0.917	0.686		
(11) Written symbol digit modality	*r*	-0.010	-0.059	-0.224	-0.026	0.100	-0.005	0.277	0.075	0.197	0.082	1.000
	*p*-value	0.941	0.668	0.097	0.850	0.463	0.972	0.039	0.581	0.146	0.546	
(12) Oral symbol digit modality	*r*	-0.201	-0.230	-0.097	0.026	0.365	0.176	0.421	0.165	0.054	0.248	0.595
	*p*-value	0.138	0.088	0.476	0.849	0.006	0.195	0.001	0.225	0.693	0.066	0.000


### Discussion

In Experiment 1, analysis of correctly reproduced grammatical and ungrammatical sequences indicated that participants showed evidence of learning both AD and NAD in both visuo-verbal and visuo-spatial tasks. Thus, adults appear capable of learning AD and NAD concurrently within the same sequence in both visuo-spatial and visuo-verbal domains with only a single, brief exposure session, even when NAD are not highlighted and when AD and NAD are composed of the same elements. Furthermore, levels of learning were better overall for AD relative to NAD, though there was no difference in levels of learning between the visuo-spatial and visuo-verbal tasks. Although it is possible there was transfer or other carryover effects from the visuo-spatial task to the visuo-verbal task, correspondence between spatial location or syllable and the grammar element, represented by “A,” “B,” etc. in the sequences in Appendix A, was randomized for each task for each participant to minimize that likelihood.

To our knowledge, this is the first time concurrent learning of AD and NAD has been demonstrated within visually presented sequences (patterns presented in a true serial, one-at-a-time format with each sequence containing both AD and NAD). Our tasks represented a particularly stringent test of participants’ abilities to simultaneously learn AD and NAD because exposure took place during one relatively brief session and the elements making up both types of dependencies came from the same set of items. Thus, neither type of dependency was highlighted in any way, and ecological validity was increased over previous tasks, in which elements composing AD and NAD have come from distinct stimulus sets (which is not the case in for example, natural language). By its nature, the reproduction task encouraged participants to deeply process each element in the sequence in the correct order, so that the sequence as a whole could be recalled. In this way, learning the AD and NAD helps participants to accomplish the recall task in a way that learning dependencies would not appear to help in the cover task of Pacton and Perruchet (2008) and Pacton et al. (2015).

Of particular note, the results also revealed interesting relationships among the different types of learning and cognitive and language abilities; however, given the exploratory nature of the correlational analyses, the relationships must be interpreted cautiously. Consistent with some previous research (e.g., Conway et al., 2010, 2011; Kidd and Arciuli, 2016), we found significant positive correlations between sequential learning and a measure of language ability, Sentence Completion, but only for the visuo-verbal non-adjacent dependency measure. Importantly, this correlation controlled for general IQ, which included expressive vocabulary and matrix reasoning skills. This finding suggests that, although there is a relationship between visual sequential learning and language, it appears to be the case that at least by adulthood, it is with performance on the more difficult or distal aspects of sequential learning (i.e., NAD) that are positively associated with language ability. This may partially be a function of the fact that by adulthood language use is so advanced that simpler forms of sequential learning (i.e., AD learning) may have little relevance. It could also be due to the language measure chosen which may require more advanced skills than other measures, given that Sentence Completion required explicit understanding of both semantic and grammatical context of a sentence, as well as robust and flexible word retrieval skills.

We also found visual-spatial selective attention and inhibition (measured with the Flanker task) to be positively related to one learning score (visuo-spatial AD). Individuals who performed better on selective attention demonstrated better spatial adjacent learning. This may suggest that, in order to perform at a comparable level on the visuo-spatial AD task (relative to the visual-verbal AD task), it may require additional visual spatial selective attention and inhibition skills. There is some evidence to suggest that visual-spatial processing may tap into executive function more so than does verbal memory (Miyake et al., 2001). Whereas the visual-spatial adjacent learning score was positively related to visual-spatial selective attention and inhibition, the verbal adjacent learning score only showed a slight trend toward a positive correlation with selective attention: *r*(54) = 0.186, *p* = 0.169. However, the two non-adjacent scores both demonstrated a significant negative relationship with attention span. This result seems inconsistent with de Diego-Balaguer et al.’s (2016) theoretical suggestion that NAD learning, as opposed to AD learning, is more dependent upon selective attention. However, it could be that because our task is characterized by incidental learning, with no apparent instruction to look for patterns, and thus from the participants’ perspective, no benefit to learning the NAD, that participants’ selective attention was devoted to doing other aspects of task performance (such as serial recall) and this allocation of attention detracted from the learning of NAD. Thus, from a certain point of view, this correlation result actually supports de Diego-Balaguer’s et al. (2016) proposal that selective attention and NAD learning are linked.

Relatedly, it is possible that because AD are more easily learned, they might become more explicit with continued exposure, even within a single exposure session, and are more likely to be within the focus of attention. Learning of NAD may, on the other hand, remain implicit for a longer period or indefinitely. Our understanding of this nuanced relationship would benefit from further exploration using additional attentional and AD and NAD learning measures, as well as experimental manipulations, to selectively promote or attenuate attention during learning. Measures of online learning would also be helpful in fleshing out subtleties of the relationship.

Interestingly, non-adjacent performance on both tasks was negatively correlated with the span scores in opposite domains (e.g., spatial sequential learning negatively correlated with verbal attention span, and verbal sequential learning negatively correlated with spatial attention span). This dissociation may suggest that having attention and memory skills in one domain (e.g., verbal) may actually interfere with learning in a different domain (e.g., spatial) and vice-versa. Interference between verbal and spatial processing has been observed for other memory tasks (e.g., Jones et al., 1995; Saults and Cowan, 2007; Morey and Miron, 2016). In turn, this finding is not inconsistent with conceptualizations of statistical-sequential learning, as having a modality-specific locus (e.g., Conway and Christiansen, 2005, 2006). Again, further experimental work is necessary to fully understand this pattern of results.

To explore how additional experience with the dependencies might differentially affect learning of the two types of patterns for the two types of tasks, we conducted Experiment 2. A subset of participants came in for a second session and engaged in all phases of the visuo-spatial and visuo-verbal sequential learning tasks. Participants also completed a new set of domain-specific working memory measures.

## Experiment 2

### Materials and Methods

#### Participants

Twenty (9 female, age range 18–29, *M* = 23) of the participants who completed Experiment 1 met criteria to participate in a follow-up fMRI study (findings not reported here) and signed up to participate. Participation in Experiment 2 was an average of 9 days after Experiment 1. All participants were paid $50. Two additional participants (both female) from Experiment 1 also participated in Experiment 2 but were excluded from analyses due to missing data caused by a computer malfunction. All participants gave written informed consent in accordance with the Declaration of Helsinki. All procedures were approved by the Center for Advanced Brain Imaging institutional review board.

#### Materials

The same visuo-spatial and visuo-verbal sequential learning tasks were used for Experiment 2. In addition, participants completed the Shortened Operation Span and Symmetry Span tasks (Foster et al., 2015), which are assessments of visuo-verbal and visuo-spatial working memory, respectively. We added these tasks because the Backward Digit Span used in Experiment 1 has been considered to not only require working memory but to include a heavy immediate memory component (e.g., Hill et al., 2010). The Shortened Operation and Symmetry Span tasks have been found to be more valid and are more reliable measures of working memory (e.g., Conway et al., 2005; Foster et al., 2015). For the Operation Span task, the participants viewed sequences of 3–7 letters to be remembered and correctly sequenced later. After each letter was presented and before the next one was presented, the participant was required to complete a math problem while holding the current sequence in mind. The symmetry span followed the same procedure except that the to-be-remembered sequence was made up of red squares in different locations on a grid, and the distractor task was to make judgments regarding whether shape displayed was symmetrical along it’s vertical axis. Symmetry span sequences were 2–5 items in length. All four computer tasks were presented on a 12.5 inch Lenovo Thinkpad laptop using Eprime 2.0.

In addition, at the end of the study, participants were interviewed about their awareness of the existence of underlying patterns in the sequential learning tasks. The interview questions were focused on participants’ subjective perception of the existence of patterns and of their confidence in their performance. Interview questions are listed in Appendix B.

#### Procedure

After completing informed consent and fMRI screening for Experiment 2, participants were taken to a private room and seated in front of the computer. They were given the same instructions as in Experiment 1 and completed the same mapping and exposure phases of the visuo-spatial sequence task as in Experiment 1. However, they did not complete the test phase at this point in the study. Sequences in exposure phases were presented in random order but were the same sequences from Experiment 1. Following the visuo-spatial task, they were given instructions and they completed the same mapping and exposure phases of the visuo-verbal sequence task from Experiment 1. Again, they did not complete the test phase and although sequences within each section were presented in random order, they were the same sequences as in Experiment 1. Next, they participated in the fMRI experiment, which included familiarity judgments of previously viewed grammatical and ungrammatical sequences from the spatial and verbal tasks (results not reported here). On each trial of familiarity judgment completed in the fMRI scanner, participants were presented with either a previously viewed grammatical or previously viewed ungrammatical sequence and asked, “Did that seem familiar?”, to which the participant responded “yes” or “no” via a button press. This task was designed for as little contamination as possible of performance on the following test phases reported here, and thus, there was no mention to participants of grammatical rules and no explicit judgment of grammaticality on the part of the participants, and despite the familiarity judgment, all sequences had previously been viewed an equal numbers of times. After their fMRI scan, participants were interviewed about their scanner experience and then completed the test phases of first the spatial task followed by the verbal task. They were interviewed again about their awareness of patterns in the sequences, and finally, completed first the Operation Span and then the Symmetry Span.

### Results

To determine whether the subset of participants who went on to participate in Experiment 2 differed on any measures from the rest of the participants from Experiment 1, a series of independent sample *t*-tests were conducted on sequential learning and cognitive measures, comparing the two groups (Experiment 1 only versus those who participated at both time points). The two groups did not differ significantly on any measures.

#### Time × Task × Adjacency

**FIGURE 6:**
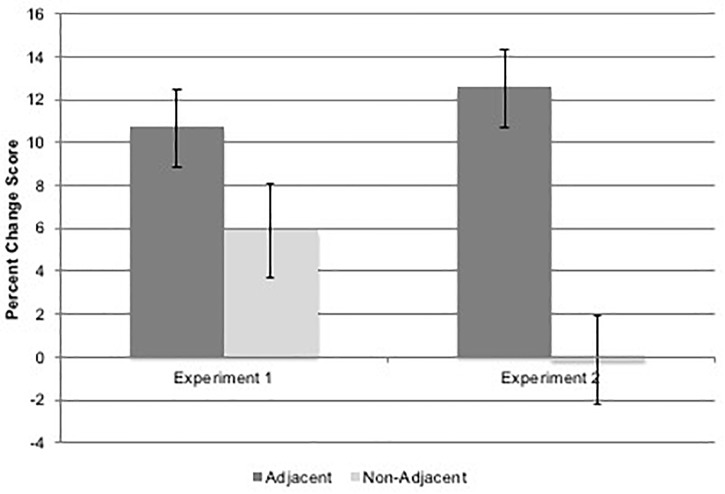
Figure shows the Experiment 2 significant (*p* = 0.037) time × adjacency interaction effect on percent change scores as a measure of learning. Error bars represent standard errors.

To examine learning differences across time and condition, a 2(Time: Experiment 1 or 2) × 2(task: spatial or verbal) × 2(adjacency) repeated measures ANOVA was conducted on percentage change scores. Results indicated a non-significant trend for main effect of time, *F*(1,19) = 3.30, *p* = 0.085, η^2^= 0.148, in which percentage change between grammatical and ungrammatical sequence scores was slightly lower at Time 2 (*M* = 6.18%, *SD* = 10.67) than at Time 1 (*M* = 8.27%, *SD* = 12.09). There was no significant main effect of task, *F*(1,19) = 1.52, *p* = 0.233, but there was the expected significant main effect of adjacency, *F*(1,19) = 9.95, *p* = 0.005, η^2^ = 0.344, in which participants showed a greater learning effect for adjacent items (*M* = 11.58%, *SD* = 14.10), compared to non-adjacent items (*M* = 2.87%, *SD* = 11.35). Finally and most interestingly, there was a significant time × adjacency interaction (see Figure 6), *F*(1,19) = 5.02, *p* = 0.037, η^2^ = 0.209).

Follow-up pairwise comparisons with a Sidak adjusted *p*-value of 0.013 showed a significant difference between adjacent and non-adjacent percentage change scores at Time 2 (*p* = 0.001) but not at Time 1 (*p* = 0.168), as well as a significant decrease in non-adjacent percentage change from Time 1 to Time 2 (*p* = 0.014) but no significant change from Time 1 to Time 2 for the adjacent percentage change (*p* = 0.356). This indicates that while learning of AD remained robust over time, learning decreased for NAD despite increased opportunity for learning. This was also born out in absolute percentage correct for each type of dependency in each task: spatial grammatical adjacent percentage correct *M* = 70.%, *SD* = 24, spatial ungrammatical adjacent *M* = 62%, *SD* = 23, spatial grammatical non-adjacent *M* = 69%, *SD* = 25, spatial ungrammatical non-adjacent *M* = 67% *SD* = 23, verbal grammatical adjacent *M* = 72%, *SD* = 17, verbal ungrammatical adjacent *M* = 64%, *SD* = 18, verbal grammatical non-adjacent *M* = 69%, *SD* = 19, and verbal ungrammatical non-adjacent *M* = 69%, *SD* = 17.

#### Correlations With Cognitive Assessments

We conducted partial correlations controlling for IQ between the percentage change scores for Experiment 2 and the two new working memory measures, Operation Span and Symmetry Span, as well as the cognitive measures from Experiment 1. Partial correlations were not adjusted for multiple correlations and the sample size for Experiment 2 was slightly underpowered for finding partial correlations, so results should be interpreted with caution. Partial correlation results indicated some interesting patterns (see Table 5 for full correlation matrix). NAD learning (but not AD learning), in both visuo-spatial and visuo-verbal domains, was significantly positively correlated with the language measure, Sentence Completion [visuo-spatial: *r*(17) = 0.575, *p* = 0.010; visuo-verbal: *r*(17) = 0.503, *p* = 0.028]. AD measures were both significantly negatively correlated with working memory measures in the opposite domain: visuo-spatial AD with Operation Span (verbal working memory measure), *r*(14) = -0.692, *p* = 0.006 (three participants were missing Operation Span data due to computer failure, thus the lower degrees of freedom), and visuo-verbal AD learning with Symmetry Span (spatial working memory measure), *r*(17) = -0.735, *p* < 0.0001. AD and NAD learning within the same modality, visuo-verbal, were significantly negatively correlated, *r*(17) = -0.710, *p* = 0.001, while conversely, NAD learning in opposite domains (visuo-verbal vs. visuo-spatial) were significantly positively correlated, *r*(17) = 0.584, *p* = 0.009. Finally, visuo-spatial NAD learning was significantly positively correlated with Oral Symbol Digit Modality Test, a test of processing speed, *r*(17) = 0.619, *p* = 0.005.

**Table 5:** Partial Correlations between Experiment 2 sequential learning measures and cognitive measures controlling for IQ (*n* = 19, Operations Span *n* = 16).

Variables		1	2	3	4	5	6	7	8	9	10	11	12	13
(1) Spatial adjacent percent change	*r*	1.000												
	*p*-value													
(2) Spatial non-adjacent percent change	*r*	0.043	1.000											
	*p*-value	0.860												
(3) Verbal adjacent percent change	*r*	0.010	-0.327	1.000										
	*p*-value	0.967	0.172											
(4) Verbal non-adjacent percent change	*r*	0.094	0.584	-0.710	1.000									
	*p*-value	0.701	0.009	0.001										
(5) Sentence completion	*r*	-0.124	0.575	-0.185	0.503	1.000								
	*p*-value	0.612	0.010	0.447	0.028									
(6) Forward digit span	*r*	0.648	0.055	-0.044	0.185	-0.164	1.000							
	*p*-value	0.003	0.823	0.858	0.448	0.501								
(7) Backward digit span	*r*	0.483	0.102	-0.168	0.137	-0.074	0.118	1.000						
	*p*-value	0.036	0.679	0.493	0.575	0.763	0.630							
(8) Forward spatial span	*r*	-0.017	0.192	0.088	-0.015	0.472	0.076	-0.293	1.000					
	*p*-value	0.944	0.432	0.720	0.950	0.041	0.756	0.223						
(9) Backward digit span	*r*	0.174	0.089	-0.208	0.244	0.127	0.141	0.028	0.026	1.000				
	*p*-value	0.476	0.717	0.392	0.314	0.603	0.565	0.909	0.916					
(10) Written symbol digit modality test	*r*	0.420	0.088	-0.280	0.152	-0.101	0.255	0.607	-0.268	0.012	1.000			
	*p*-value	0.074	0.720	0.246	0.536	0.682	0.292	0.006	0.268	0.960				
(11) Oral symbol digit modality test	*r*	-0.277	0.619	-0.357	0.339	0.560	-0.517	0.039	0.099	-0.006	0.140	1.000		
	*p*-value	0.251	0.005	0.134	0.155	0.013	0.024	0.873	0.686	0.980	0.568			
(12) Flanker	*r*	-0.217	-0.067	0.001	0.058	-0.018	-0.170	0.170	-0.240	0.591	-0.055	-0.003	1.000	
	*p*-value	0.372	0.786	0.995	0.812	0.940	0.487	0.485	0.322	0.008	0.823	0.990		
(13) Operations span	*r*	-0.692	-0.062	0.167	-0.243	0.202	-0.549	-0.203	0.255	0.052	-0.570	0.142	0.546	1.000
	*p*-value	0.006	0.821	0.536	0.364	0.454	0.028	0.451	0.341	0.849	0.021	0.600	0.029	
(14) Symmetry span	*r*	0.311	0.094	-0.735	0.383	-0.063	0.333	0.329	0.003	0.363	0.164	-0.087	0.119	-0.149
	*p*-value	0.196	0.702	0.000	0.105	0.798	0.164	0.168	0.989	0.126	0.503	0.724	0.628	0.582


#### Correlations With Measures of Pattern Awareness

Participants reported moderate levels of pattern awareness with a mean spatial pattern level score of 3.45 (*SD* = 1.02, range = 1–5) out of 5 (5 highest, Appendix B question 3) and a mean verbal pattern level score of 3.30 (*SD* = 0.87, range = 1–5, Appendix B question 4). Thus, for both tasks, participants’ level of awareness on average fell somewhere between “there may have been a pattern” and “there was a pattern at certain times.” In addition, 50% of participants endorsed that there was “more than one pattern” in the spatial task while 45% endorsed it in the verbal task (Appendix B, question 5). On average, those participants who reported consciously noticing patterns, noticed them in both tasks during the training phase of Experiment 2, and thus, did not notice them during Experiment 1. Participants were somewhat confident of their reproduction of both spatial sequences (mean rating of 6.17 out of 10) and verbal sequences (mean rating of 5.94). Finally, for both the spatial and verbal tasks, only 20% (4 participants) endorsed that sometimes there were mistakes in the sequences.

To determine whether pattern perception and confidence were related to sequential learning performance, interview question scores were correlated with each of the sequential learning percent change scores resulting in the following significant correlations between interview questions and sequential learning. Point-biserial correlations indicated that both “noticing mistakes in the spatial sequences” (Appendix B, question 7), *r*_pb_(20) = -0.506, *p* = 0.023, and “noticing mistakes in the syllable sequences”, *r*_pb_(20) = -0.506, *p* = 0.023, were significantly negatively correlated with visuo-spatial AD percentage change learning score. Kendall’s Tau correlations indicated marginally significant negative correlations between spatial pattern awareness level (Appendix B, question 3) and visuo-spatial NAD learning, τ(20) = -0.315, *p* = 0.078. Verbal pattern awareness level (Appendix B question 4) was marginally significantly negatively correlated with visuo-verbal AD τ(20) = -0.399, *p* = 0.063.

### Discussion

The same adjacency effect found in Experiment 1 was also present in Experiment 2, indicating that AD was learned better than NAD. Interestingly, there was also a significant time by adjacency interaction, revealing that there was a decrease in learning from Experiment 1 to Experiment 2 for NAD but no change in learning for AD. This suggests that although adults are capable of learning both AD and NAD within the same visuo-spatial and visuo-verbal sequences, the learning of AD may be more impervious to disruption by counter examples (continued exposures to ungrammatical sequences) or other explicit decision tasks. That is, by the time of the second test phase in Experiment 2, participants had been exposed to a set of ungrammatical sequences from the Experiment 1 test phase and from the fMRI familiarity task. There were certainly many more exposures to grammatical sequences than ungrammatical, due to the exposure phases in sessions 1 and 2, but by the second test, participants had also seen a number of counter examples to the grammar. Specifically, about a quarter of the sequences did not follow the grammar, making the grammatical regularities probabilistic rather than deterministic. The learning of NAD appears less robust in this sense, less able to detect the consistent structure in noisy data. A more stable structure, one that continued to be deterministic (100% predictive), may have allowed for NAD regularities to become more crystallized by giving more exposure to the rules without interference from the unreliable and non-predictive noise of violations. Thus, the better learning of AD may be the result of AD being available for learning sooner through a more implicit system, which may come online earlier in the task and therefore allow AD to be learned faster.

A chunking account could also potentially explain differences in the AD and NAD learning over time. Chunking has been proposed to be a mechanism for learning sequential and statistical regularities in implicit learning tasks (e.g., Perruchet and Pacton, 2006; Page and Norris, 2009). The idea is that adjacent stimuli are grouped together into chunk representations and receive increased activation every time that the same chunk is subsequently encountered. As learning proceeds, chunk representations become more order-sensitive and begin to compete with each other (Page and Norris, 2009; Smalle et al., 2016). This competition process means that when the same individual stimuli are encountered in other sequences but not part of the same previously encountered chunk (e.g., ungrammatical sequences), this can attenuate learning and make it harder to distinguish the original chunked sequence from other sequences containing the same stimuli but in different sequential arrangements (Page et al., 2013; Smalle et al., 2016). Thus, although a chunking account can potentially explain the interference that ungrammatical sequences provide, it does not necessarily disambiguate why the learning and representation of NADs would suffer more so than ADs. We suggest it is likely that chunking processes are more sensitive to ADs compared to NADs, due to the mechanism of chunking itself, which focuses on forming representations of adjacent stimuli, thus making NAD learning more fragile.

On the other hand, another way to interpret these results is that learning of NAD may be more flexible and more able to incorporate new information; that is, through experience with the non-adjacent violations, participants assimilated the ungrammatical regularities, leading to behavioral facilitation (improved recall) for the ungrammatical sequences. This can be thought of as learning occurring for the ungrammatical patterns. This effect was not observed for adjacent violations.

A final possibility is that participants experienced negative transfer for AD but not for NAD. Woltz et al. (2000) found that high-skill participants, those who had been given a large amount of practice processing a particular set of sequences, experienced interference from the well-remembered sequences that caused a higher level of errors on a new set of sequences. The new sequences, like our ungrammatical sequences, began like the familiar sequences but ended differently, similarly to the violations in our ungrammatical sequences. Low-skill participants, those who had much less practice with the original sequences, did not experience as much negative transfer. In addition, negative transfer errors persisted longer for those with more practice. It is possible that increased level of practice represents “better learning.” If that is the case, it could be that because ADs were learned better than NADs in this study, participants showed more persistent errors in ungrammatical AD sequences than in ungrammatical NAD sequences. As the AD errors in ungrammatical sequences persisted into Experiment 2 while the NAD errors did not, the greater difference between performance on grammatical and ungrammatical AD sequences showed a continued high level of learning. On the other hand, by Experiment 2, the errors in the ungrammatical NAD sequences had dropped off leaving a smaller difference between AD and NAD performance reflecting the lower learning of NAD. Further research is required to flesh out the mechanisms behind the drop-in learning of NAD from Experiment 1 to Experiment 2.

The correlation results identified in Experiment 2 include findings that are complementary to those revealed in Experiment 1. Specifically, once again there were negative correlations between sequential learning and working memory capacity in the opposite domain. That is, visuo-spatial sequential learning (of adjacent dependencies) was negatively correlated with Operation Span, a measure of visuo-verbal working memory, and visuo-verbal sequential learning (of adjacent dependencies) was negatively correlated with Symmetry Span, a measure of visuo-spatial working memory. In addition, in the context of Experiment 2, an interesting positive correlation between visuo-spatial and visuo-verbal non-adjacent sequential learning was observed, suggesting that learning of NAD across domains may rely on similar cognitive processes and/or learning strategies. Additionally, there was a significant negative correlation between visuo-verbal adjacent and non-adjacent learning. This suggests that one’s ability to learn one type of dependency may have a slight inhibitory effect on learning the other type, perhaps because sensitivity to one type masks the other as suggested by the findings from Gómez (2002) and Romberg and Saffran (2013).

Finally, participants appeared to display a moderate subjective perception that patterns were present in the stimuli and a few participants may even have consciously noticed violations to those patterns. However, those participants who perceived that there were sometimes mistakes in either the visuo-verbal or visuo-spatial sequences showed lower levels of learning on visuo-spatial adjacent dependencies. This seems inconsistent with results from Experiment 1 showing a positive correlation between level of attention and learning of spatial adjacent dependencies. However, continued high levels of attention after consolidation of Experiment 1 learning could potentially lead attention to shift from what has already been learned (spatial AD) to violations of those dependencies. Noticing those violations could in turn allow participants to recall the sequences with violations more accurately. In such cases, participants would show “lower learning” not by failing to take advantage of the boost to grammatical performance that recognizing AD can give, but by boosting performance on ungrammatical sequences to be more like that of grammatical sequences. It is also plausible that the explicit familiarity judgment during the fMRI task changed the relationship of which cognitive resources are most associated with better performance on sequential learning. However, this explanation seems unlikely given that great care was taken not to mention to participants that grammatical rules existed within sequences, and thus, it is unlikely that they were making explicit judgments about AD and NAD, but rather, simply about familiarity.

## General Discussion

This study investigated the learning of AD and NAD presented in two different (visual) domains: spatial and verbal. To our knowledge, this study provides the first evidence that adults can learn visual serially presented AD and NAD concurrently within the same sequences. Not only that, but they were able to learn the NAD across more than a single intervening item, which has rarely been tested. Consistent with auditory studies (e.g., Gómez, 2002; Romberg and Saffran, 2013), AD was learned better than NAD, but there was no difference between visuo-spatial and visuo-verbal sequences. Experiment 1 also showed that NAD learning was significantly correlated with a language measure, Sentence Completion, even after controlling for IQ performance. This suggests a specific relationship between NAD learning and an advanced language skill involving semantic and syntactic knowledge, as well as predictive ability, stemming, perhaps, from similar underlying processes.

Experiment 2 further showed that with continued exposure to both grammatical and ungrammatical sequences, learning decreased, but only for the NAD. This suggests that NAD learning was more fragile, similar to findings of Romberg and Saffran (2013), who suggested that AD is learned implicitly, and thus, may be more robust while NAD is learned explicitly. It is also possible that NAD learning was earlier in its learning trajectory than AD learning, and so when ungrammatical sequences were introduced in the test phase, participants may still have been flexibly incorporating new information, including ungrammatical sequences, into their representations of the regularities. In addition, negative transfer (Woltz et al., 2000) might also have been at work for the better learned AD, keeping the difference between ungrammatical and grammatical larger, again showing stronger learning of AD. A chunking account may also offer insights into the nature of competition and interference derived from being exposed to ungrammatical sequences (Page et al., 2013), though it is not clear whether such an account can explain why interference was observed for the learning and representation of NADs but not ADs. Analyses of errors in future studies might help in further exploring whether these mechanisms are involved by determining whether errors made in some grammatical sequences followed patterns in ungrammatical sequences (as in flexibly incorporating new information), errors in ungrammatical sequences followed patterns in grammatical sequences (as in negative transfer), or errors involve making chunks where they were not presented.

Both Experiments 1 and 2 were consistent in showing negative correlations between sequential learning in one domain (e.g., spatial or verbal) and working memory or attention span in the other domain. This finding suggests that to some extent, learning and executive function processes in different domains may have a competitive or inhibitory relationship with one another. Kirby (1993) suggested that although verbal and spatial learning strategies can complement each other when an individual has adequate abilities in both, when abilities in the two domains are out of balance, they may have competitive effects on each other. There may be similar competitive effects here. Although this outcome was found across both experiments with different measures of similar constructs, again, replication with a larger, independent sample is necessary to confirm this result.

Furthermore, while there was a positive correlation between spatial and verbal NAD learning, there was a negative correlation between AD and NAD for verbal sequences. This may indicate that to some extent, NAD learning relies on similar underlying cognitive processes or strategies, even across different domains, whereas for learning of AD and NAD, different processes are at work and may even interfere with each other. This latter finding is again consistent with those of Romberg and Saffran (2013), who showed that the learning of AD may rely on more implicit learning and be more robust while learning of NAD may involve more explicit learning and be more fragile. Conway et al. (unpublished) showed that learning of AD and NAD involves distinct brain networks. Learning AD involved a distributed network of occipital and frontal brain regions that likely mediate perceptual, attention, and working memory operations, whereas the learning of non-adjacent dependencies crucially relied on the anterior cingulate cortex, which is thought to mediate cognitive control and inhibition functions.

In summary, these results suggest that adults have an impressive facility, incidentally encoding both adjacent and non-adjacent visual patterns across both spatial and verbal stimuli. However, two lines of evidence suggest that the cognitive processes underlying the learning of AD and NAD are at least partially dissociable and may change over repeated exposure. First, AD learning was more robust than NAD learning across multiple sessions, with NAD learning declining over time. Second, the pattern of correlations indicated that NAD learning ability may be similar across verbal and spatial domains, whereas learning NAD and AD may be different, even within the same domain, and may even interfere with each other. Research employing neuroimaging methods, such as fMRI and event-related potentials (ERP), is currently underway to further our understanding of the neurocognitive mechanisms underlying sequential processing of proximal and distal events in different cognitive and perceptual domains. This work will help to further disambiguate common and distinct mechanisms involved in learning adjacent and non-adjacent patterns in language and other complex skills.

## Ethics Statement

This study was carried out in accordance with the recommendations of the institutional review board of the Center for Advanced Brain Imaging with written informed consent from all subjects. All subjects gave written informed consent in accordance with the Declaration of Helsinki. The protocol was approved by the institutional review board of the Center for Advanced Brain Imaging.

## Author Contributions

CC and TK contributed to conception of the study. JD, CC, and TK contributed to the design of the study. JD collected the data, performed the statistical analysis, and wrote the first draft of the manuscript. All authors contributed to manuscript revision, read and approved the submitted version.

## Conflict of Interest Statement

The authors declare that the research was conducted in the absence of any commercial or financial relationships that could be construed as a potential conflict of interest.

## Footnotes

1 This method of creating sequences did result in a large number of sequences with repetitions within the sequence which might be expected to cause those particular sequences to be more easily recalled. However, repetitions were incidental to the grammatical rules and could not help participants to learn AD or NAD. Repetitions were evenly distributed across AD (e.g., 20 AD sequences with simple repeats of at least one item presented twice in a row within the sequence, 12 grammatical AD repeats and 8 ungrammatical AD repeats) and NAD (e.g., 19 simple repeats, 12 grammatical NAD repeats and 7 ungrammatical AD repeats) sequences. Although there was a difference in the number of grammatical and ungrammatical sequences containing repetitions, when analyses were done using only sequences with no repetitions, results were the same as when the entire data set was used (see Experiment 1 “Results”).

## Appendix A

### List of Grammatical and Ungrammatical Test Sequences

**Table A1:** Below are the grammatical and ungrammatical sequences used for testing. Ungrammatical sequences were made by changing two elements (in bold) in a grammatical sequence.

Grammatical sequences	Ungrammatical sequences
	Adjacent ungrammatical
AADBCBC	AA**B**BC**D**C
ABABDCC	AB**C**BD**A**C
ACBBBAC	AC**A**BB**C**C
ADCBDCC	AD**A**BD**B**C
BADCDCD	BA**C**CD**A**D
BBACCBD	BB**D**CC**A**D
BCBCDCD	BC**D**CD**B**D
BDCCBAD	BD**B**CB**D**D
CADDADA	CA**B**DA**C**A
CBADBAA	CB**C**DB**D**A
CCBDADA	CC**A**DA**B**A
CDCDCBA	CD**A**DC**D**A
DADABAB	DA**C**AB**C**B
DBAAADB	DB**D**AA**C**B
DCBACBB	DC**D**AC**A**B
DDCAADB	DD**B**AA**B**B
	
	Non-adjacent ungrammatical
AADBADC	AAD**C**AD**A**
ABABBAC	ABA**D**BA**B**
ACBBADC	ACB**D**AD**B**
ADCBCBC	ADC**C**CB**B**
BADCBAD	BAD**D**BA**B**
BBACADD	BBA**A**AD**C**
BCBCCBD	BCB**A**CB**C**
BDCCADD	BDC**D**AD**C**
CADDCBA	CAD**A**CB**C**
CBADDCA	CBA**B**DC**D**
CCBDBAA	CCB**B**BA**D**
CDCDDCA	CDC**A**DC**D**
DADADCB	DAD**B**DC**D**
DBAACBB	DBA**C**CB**A**
DCBADCB	DCB**C**DC**A**
DDCABAB	DDC**B**BA**A**

*All grammatical sequences came from the same set. However, each grammatical sequence was used to make either an adjacent ungrammatical or non-adjacent ungrammatical sequence, and thus for analyses, was accordingly labeled as adjacent grammatical or non-adjacent grammatical despite the fact that they did not differ from each other in adjacency. In the list below, ungrammatical sequences are paired with the grammatical sequences from which they were created.*

## Appendix B

### Experiment 2 Post Test Pattern Awareness Interview Questions

(1) On a scale of 1 to 10, how confident were you in your sequence imitation for the squares task?

• 1 2 3 4 5 6 7 8 9 10

(2) On a scale of 1 to 10, how confident were you in your sequence imitation for the syllables task?

• 1 2 3 4 5 6 7 8 9 10

(3) Did you notice a pattern in the sequences presented in the squares task? If so, try to describe it.

• (5) There was definitely a pattern
• (4) There was a pattern at certain times
• (3) There may have been a pattern
• (2) The items occurred somewhat randomly
• (1) There was absolutely no pattern at all

(4) Did you notice a pattern in the syllables task? If so, try to describe it.

• (5) There was definitely a pattern
• (4) There was a pattern at certain times
• (3) There may have been a pattern
• (2) The items occurred somewhat randomly
• (1) There was absolutely no pattern at all

(5) Was there more than one pattern in either task? Explain.

• (1) No
• (2) Yes

(6) If you noticed a pattern, at what point did you notice it?

(a) **Squares task:**

• (3) During the first training session in the other lab
• (2) During the second training session here before the scanner
• (1) During the test in the scanner

(b) **Syllables task:**

• (3) During the first training session in the other lab
• (2) During the second training session here before the scanner
• (1) During the test in the scanner

(7) Did it ever seem like there were mistakes in the sequences? Squares task? Syllables task? Explain.

• (1) No
• (2) Yes
